# Impact of Cellular miRNAs on Circulating miRNA Biomarker Signatures

**DOI:** 10.1371/journal.pone.0020769

**Published:** 2011-06-17

**Authors:** Radha Duttagupta, Rong Jiang, Jeremy Gollub, Robert C. Getts, Keith W. Jones

**Affiliations:** 1 Applied Research, Affymetrix Inc, Santa Clara, California, United States of America; 2 Research and Development, Genisphere LLC, Hatfield, Pennsylvania, United States of America; University of Melbourne, Australia

## Abstract

Effective diagnosis and surveillance of complex multi-factorial disorders such as cancer can be improved by screening of easily accessible biomarkers. Highly stable cell free Circulating Nucleic Acids (CNA) present as both RNA and DNA species have been discovered in the blood and plasma of humans. Correlations between tumor-associated genomic/epigenetic/transcriptional changes and alterations in CNA levels are strong predictors of the utility of this biomarker class as promising clinical indicators. Towards this goal microRNAs (miRNAs) representing a class of naturally occurring small non-coding RNAs of 19–25 nt in length have emerged as an important set of markers that can associate their specific expression profiles with cancer development. In this study we investigate some of the pre-analytic considerations for isolating plasma fractions for the study of miRNA biomarkers. We find that measurement of circulating miRNA levels are frequently confounded by varying levels of cellular miRNAs of different hematopoietic origins. In order to assess the relative proportions of this cell-derived class, we have fractionated whole blood into plasma and its ensuing sub-fractions. Cellular miRNA signatures in cohorts of normal individuals are catalogued and the abundance and gender specific expression of bona fide circulating markers explored after calibrating the signal for this interfering class. A map of differentially expressed profiles is presented and the intrinsic variability of circulating miRNA species investigated in subsets of healthy males and females.

## Introduction

A considerable proportion of the animal genome representing both DNA and coding/non-coding RNAs can be detected in circulation. Identified first in 1948 and thought to originate as products of apoptosis or active release from cells, extracellular circulating DNA fragments ranging in size between 500 bp to greater than 30 Kb have been characterized both in normal and diseased individuals [1], [2]. Although the physiological functions of these circulating species are unclear, the presence of tumor associated genetic alterations in these molecules combined with inherent molecular stability makes them attractive substrates for disease detection, tracking and prediction. Of the various classes of circulating nucleic acids – miRNAs, characterized by highly conserved small non-coding RNAs of 19–25 nt in length and representing approximately 1–2% of the known genes in eukaryotes [3] are particularly attractive candidates. Approximately 940 mature miRNAs have been characterized to date in humans [4], [5], [6] and it is believed that approximately 30% of all annotated human genes may potentially be targeted by miRNAs through post-transcriptional mechanisms [7]. The number of targets is likely to increase when taking into account widespread unannotated transcription [8] - thus making these molecules a powerful regulatory class with the potential to intercept a wide network of fundamental cellular processes. Over the past several years an increasing number of miRNAs have been implicated in cancer development with mechanisms ranging from copy number alterations/mutations/epigenetic silencing or dysregulated transcriptional control of miRNA loci [9], [10]. These data reveal the oncogenic and tumor suppressive nature of miRNAs and highlight not only the correlation between various cancers and differential miRNA signatures but also underscores the emergence of these molecules as a critical class of diagnostic biomarkers. The ability to profile miRNAs in circulation thus represents a non-invasive opportunity to investigate disease-specific miRNAs and is a promising alternative approach to current strategies for cancer surveillance. Evidence for the involvement of secretory miRNAs in pathogenic conditions are numerous [11], [12], and range from elevated levels of hsa-miR-155, hsa-miR-21 and hsa-miR-210 in patients with diffuse large B cell lymphoma [13], over-expression of hsa-miR-141 in individuals with prostate cancer [14] to up-regulation of hsa-miR-24 and hsa-miR-31 in squamas cell carcinoma of the oral cavity [15].

A critical prerequisite for developing circulating miRNA-based diagnostics is the ability to accurately isolate and measure representative miRNA species in biofluids. In spite of high concentration of RNAses in plasma and serum, circulating miRNAs are surprisingly tractable. Some of the key molecular properties of these species include (1) high stability in circulation and the ability to survive unfavorable physiological conditions such as extreme variations in pH and multiple freeze thaw cycles [14], [16], [17], (2) can be tumor derived thus directly reflecting disease burden [11] and (3) protected from degradation though inclusion in RNA binding proteins [18] or sub-cellular particles such as exosomes or microvesicles [11], [19] distinct from the hematopoietic cellular population. All of these cellular attributes are susceptible to a variety of pre-analytic factors involving sample collection, processing, storage and extraction methods that can determine both the quantitative and qualitative effectiveness of this species for clinical use. In an effort to standardize results and bring uniformity to data quality several studies over the past few years have begun to explore and put forward recommendations for a subset of these pre-analytical variables [14], [16], [17], [20]. In this report we explore the contribution of cellular miRNAs of hematopoietic origin in the isolation and analysis of cell-free circulating miRNAs. We find considerable proportions of miRNAs derived from Red and White Blood Cells, present as contaminants in plasma preparations with the potential to mask the intensities of truly circulating miRNA species. A sample extraction pipeline is proposed that optimizes the integrity and detection of cell-free RNA while minimizing the presence of interfering cellular miRNAs. An application of this approach is demonstrated in the analysis of inter-individual variability in normal cohorts of male and female individuals. Maps of circulating miRNAs are presented after extracting out interfering signals from miRNAs of cellular origin. Furthermore gender specific signatures in the profiles of male and female individuals are discussed. Taken together these data underscore the importance of cellular miRNA signatures as yet another variable in interpreting the complexity of circulating miRNA profiles.

## Results

### 1. Fractionation of blood plasma and profiling of cellular miRNAs

**Figure 1:**
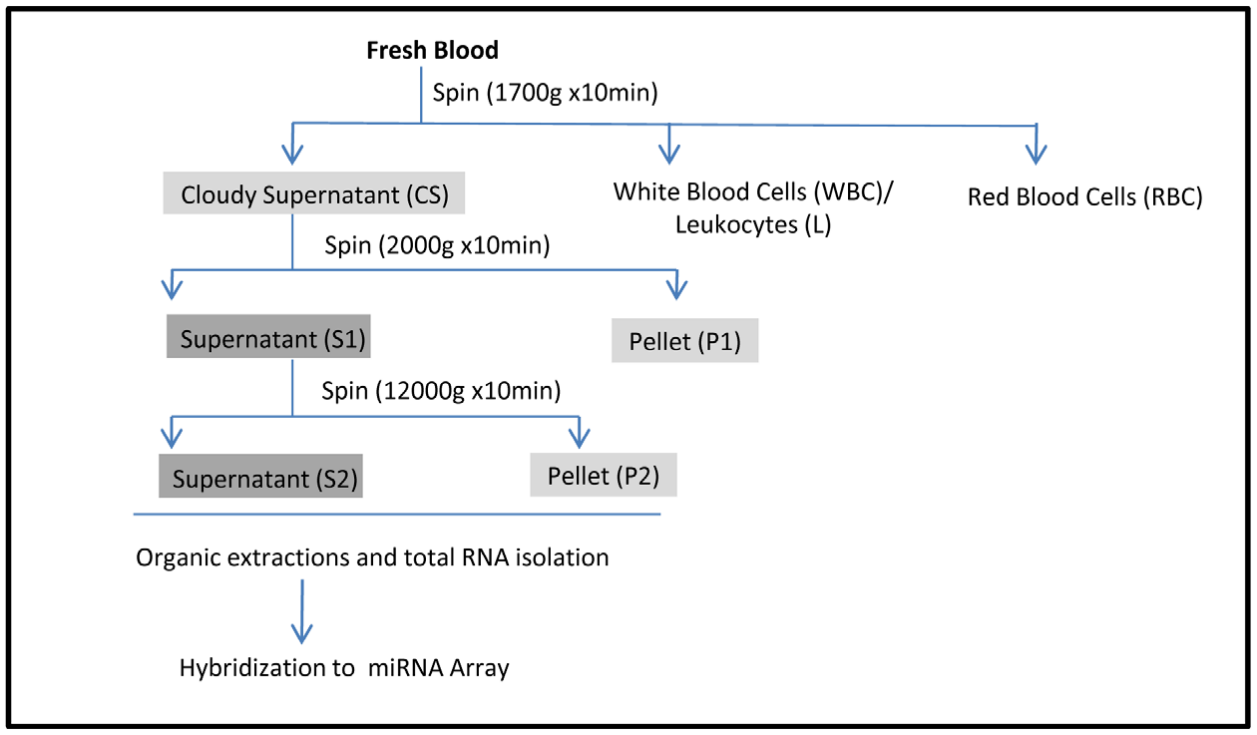
Fractionation Workflow. Separation of whole blood into distinct fractions: WBC, RBC, Leukocytes and CS, S1, S2, P1, P2 through differential centrifugation. Total RNA was extracted from each fraction and hybridized to miRNA arrays.

**Figure 2:**
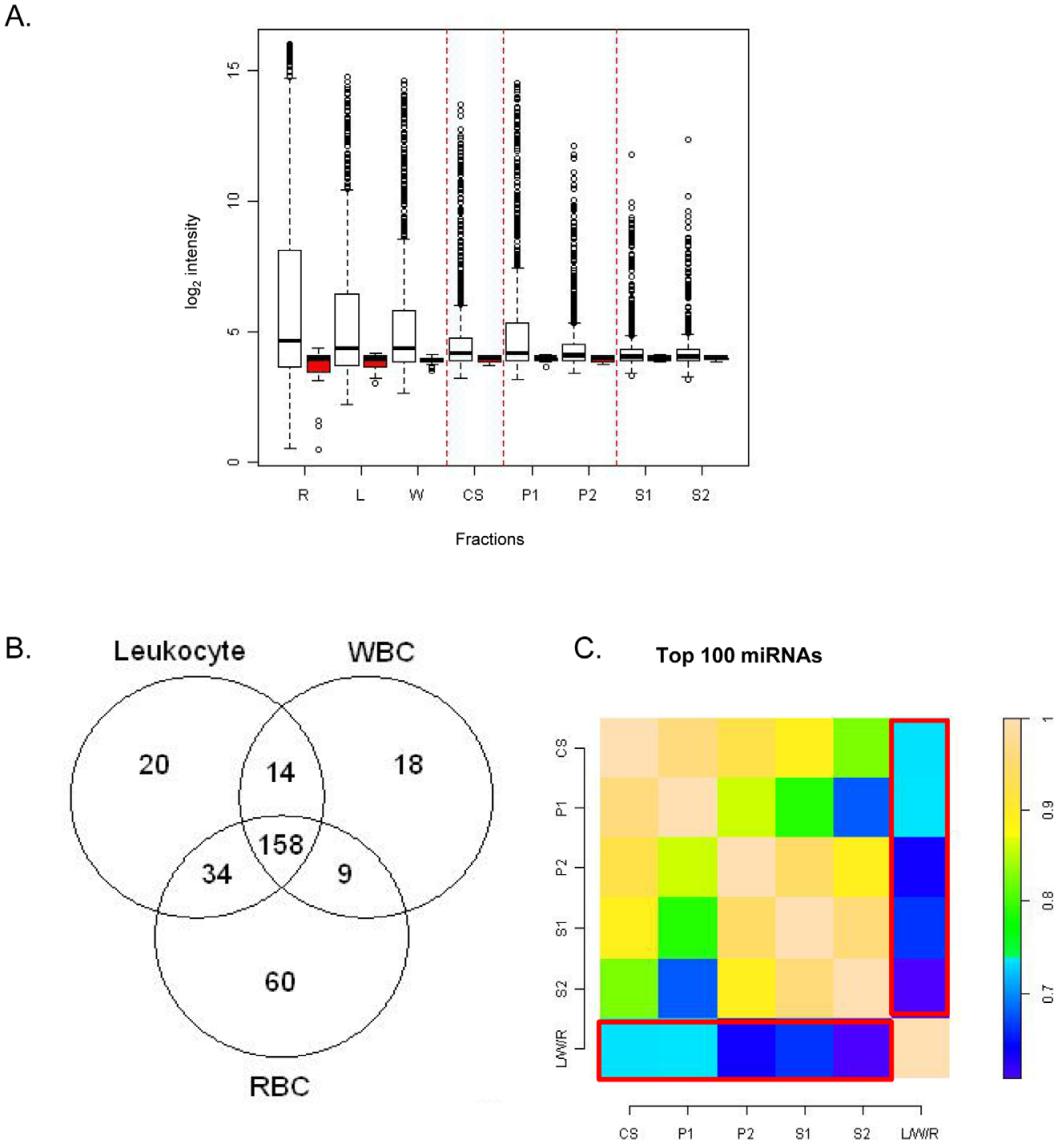
Profiling of blood derived fractions and correlation of miRNA intensities between individual fractions and contaminant class. (A) Box plots representing background subtracted non-normalized and summarized log_2_ intensities of human miRNAs (white) and background probes (red) from each fraction. The black bar represents the median of each distribution. The open circles represent the outliers. (B) Counts of detected features in Leukocytes (L), WBC (W) and RBC (R) constituting the contaminant profile. (C) Heat map of Spearman's Rank Correlation coefficients of the highest expressing 100 miRNAs across all 5 plasma fractions (CS, S1, S2, P1 and P2). The contaminant class is designated as LWR and represents 313 miRNAs derived from the union of the Leukocytes, WBC and RBC fractions. Correlation values are shown in the bar scale.

Conventional protocols for isolation of plasma (the non-cellular component of blood that remains after removal of cells by centrifugation) involves variable combinations of individual or multiple low/high speed centrifugation steps. In order to assess the contribution of these differential spin steps in the removal of cellular material, we sought to fractionate whole blood from 17 healthy male Caucasian donors (Table S1) through successive centrifugation rounds and investigate the proportion of cell free and cellular miRNAs at each stage (Fig. 1). Samples were initially segregated at a low speed spin to generate plasma (Cloudy Supernatant) along with three cellular fractions: (1) Red Blood Cells (RBCs), (2) the Buffy Coat consisting of platelets and White Blood Cells (WBCs) and (3) pure populations of leukocytes isolated by sub-fractionation of blood through Ficoll-Hypaque gradients (Leukocytes). Since these populations represent the major cellular constituents of blood, we hypothesize them to be the primary contributor of cellular material through either cell carryover or lysis during blood fractionation and collectively define them as contaminant miRNAs. We further segregated the plasma derived cloudy supernatant layer through increasing centrifugal forces into supernatant (S1 and S2) and pellet (P1 and P2) fractions. The supernatant fractions are representative of primarily cell-free circulating RNAs with the pellet fractions characterizing contaminating cellular particles. Amongst the pellet class – the P1 and P2 fractions are distinct in both size and granularity of the isolates. A prominent precipitate is distinguished in the P1 fraction while the P2 fraction has inconsequential cellular particles. All eight fractions (Fig. 1) were extracted using Trizol-LS (Invitrogen) and the mirVANA filters (Ambion) and subsequently hybridized to the Affymetrix miRNA arrays. Integrity of extracted RNA was evaluated by Bioanalyzer or Polyacrylamide Gel Electrophoresis and for all fractions samples were found to be of robust quality (Fig. S1). On analysis of the data, we observe distinct gradients in intensity distributions in all of the 5 plasma derived classes compared to the contaminant miRNAs (Fig. 2A). Fractions isolated with minimal centrifugation (Cloudy supernatant) or hypothesized to be contaminant rich (P1 and P2) display a greater magnitude of signal intensity compared to supernatant fractions of S1 and S2. Taken together these distributions indicate the presence of miRNAs of distinct abundances in the cellular and circulating class and highlights the prevalence of low-abundant species in plasma fractions clarified through successive purification steps.

### 2. Correlation of miRNA intensities between individual fractions and contaminant class

Since the process of sequential centrifugation is hypothesized to result in the segregation of cellular material, we anticipate an increased concentration of contaminant miRNAs in the pellet fraction compared to the clarified supernatant derived from each centrifugation step. Consequently we hypothesize a greater correlation in miRNA populations between the contaminating cellular RNAs and the pellet fraction, compared to the supernatant fractions of S1 and S2. In order to assess the concordance in detected miRNAs between the cellular and each of the plasma derived fractions we first computed the common set of all detected miRNAs in the three contaminant classes. A total of 313 features (Table S2) were identified through a union of WBC, RBC and Leukocytes fractions based on the number of detected features in each fraction assessed through the Wilcoxon - Rank Sum Test (Fig. 2B). These features were then used to compute Spearman's rank correlations with the top 100 highest expressing miRNAs derived from the centrifugation of whole blood into 5 plasma-derived fractions (CS, S1, S2, P1 and P2) (Fig. 2C). We observe a stronger correlation between the CS and P1 fraction with the contaminant list consistent with the hypothesis that these fractions are enriched in cell-associated miRNAs. A reduced correlation is observed for fractions derived from CS (S1, S2 and P2) indicating a progressive clarification of contaminating miRNAs through removal of cellular particles with subsequent centrifugation steps. Interestingly a strong agreement is also observed between S1 and the S1 derived S2 and P2 fractions suggesting that there is no additional advantage in sub-fractionating the S1 sample. Taken together these results indicate that there is a considerable proportion of cellular material that persist in the CS fraction of plasma after the initial spin which can be sufficiently and adequately removed by only one additional low speed centrifugation step. The supernatant fraction (S1), derived after centrifugation display low levels of contaminating miRNAs (Fig. 2C) consequently leading to an enrichment of circulating miRNA species.

### 3. Concordance in expression levels of circulating miRNA species between the plasma derived classes

**Figure 3:**
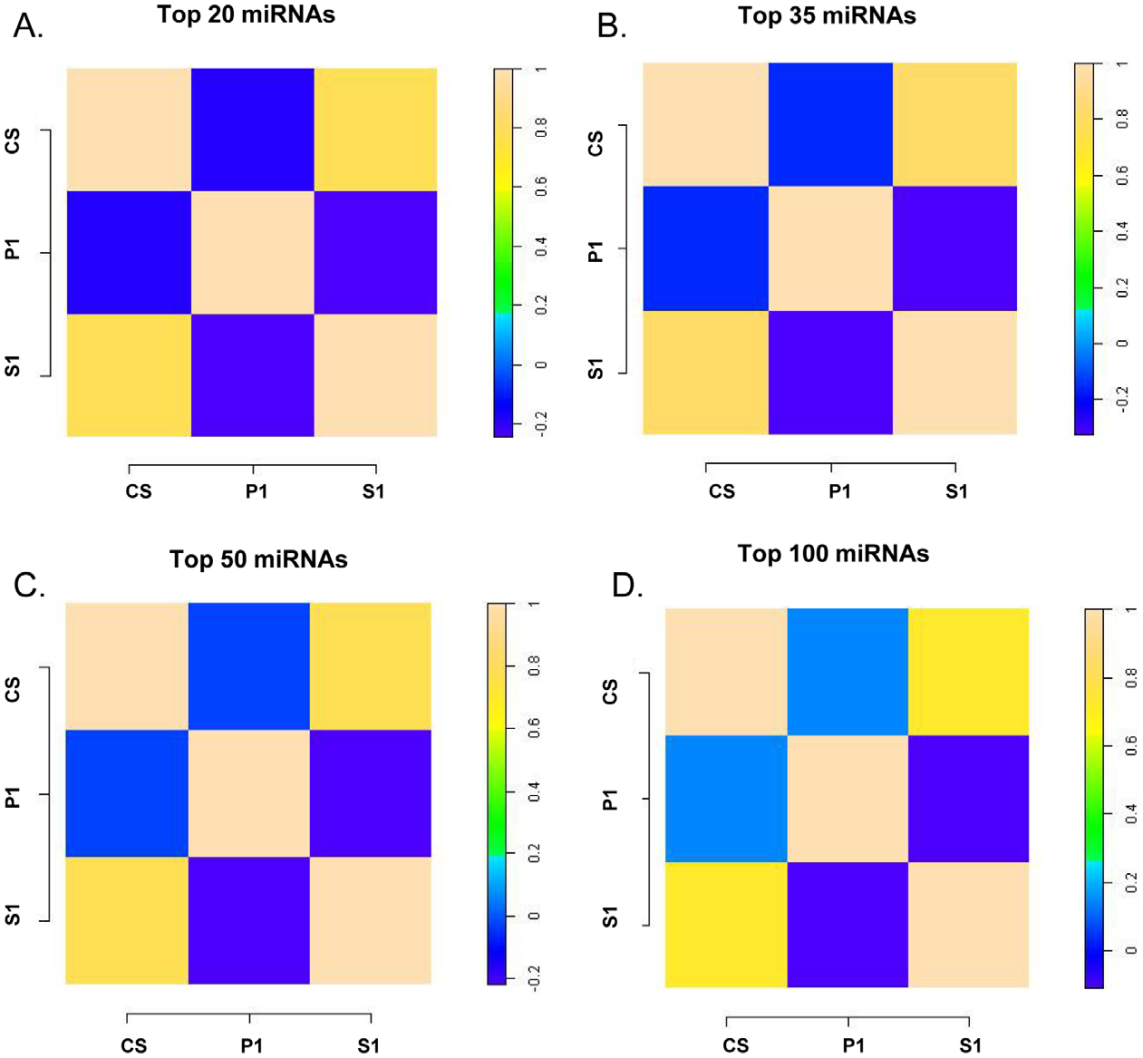
Concordance of expression levels of circulating miRNA species between the CS, S1 and P1 fractions. (A–D) Heat map of Spearman's Rank Correlation coefficients for the highest expressing 20, 35, 50 and 100 miRNAs present in CS, P1 and S1 fractions after removal of contaminant features. The correlation values are shown in the bar scale.

In order to ensure that sub-fractionation of the CS into the S1 and P1 categories did not result in the loss of true circulating miRNA species, we assessed the concordance in intensities between the CS and ensuing S1 and P1 fractions to determine their extent of similarity. Spearman's Rank Correlation analysis was performed on all detected miRNAs after removal of contaminant features in the 3 samples (CS, S1 and P1). The filtered miRNAs were first stratified into different intensity bins consisting of highest expressing 20, 35, 50 and 100 miRNAs based on average intensity levels across all fractions (Fig. 3A–D) and correlation values calculated for each pair. We observe strong and improved rank order correlations between the CS and S1 fractions (rank correlations >0.6) compared to the CS, S1 and P1 fraction pairs (rank correlations <0.0). This implies that the composition of the P1 fraction is distinct from the CS and S1 fractions and consists of unique species of miRNAs. In contrast the strong rank-order correlation between the CS and the S1 class demonstrates the presence of homogenous populations of miRNAs, indicating a preservation of miRNA species between the two plasma fractions through the fractionation process.

### 4. Comparison of expression levels of circulating and cellular miRNAs in the CS and S1 fractions

**Figure 4:**
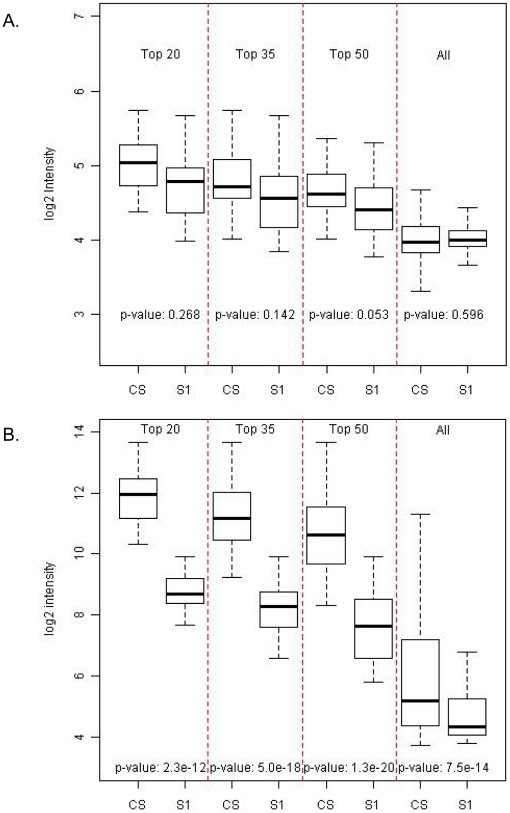
Comparison of expression levels of circulating and cellular miRNAs in the CS and S1 fractions. (A) Intensity distributions from the highest expressed 20, 35, 50 or all 534 human miRNAs in the CS and S1 fractions after removal of contaminant features. P-values from paired Student's t-tests, contrasting the intensities for each pair of conditions are reported. (B) Intensity distributions from the highest expressed 20, 35, 50 or all 313 contaminant miRNAs in the CS and S1 fractions with p-values from paired t-tests measuring significance.

Since the process of sub-fractionation may impact both the integrity and abundance of miRNAs, we wanted to ensure that markers that were conserved between the CS and the S1 fractions did not vary in their expression levels. In order to test this, categories of miRNAs that are common to both these fractions were selected after filtering for contaminants and stratified into different intensity bins consisting of the top 20, 35, 50 or all of the 534 miRNAs (total number of features that remain after removal of 313 contaminant miRNAs). We observe no significant difference in the expression levels for any of these miRNA classes in either of the supernatant fractions (two sided Student's t–test, with p-values ranging from 0.053 to 0.596) (Fig. 4A). This indicates that most miRNA species remain intact through the separation process from CS to S1. In addition, since the isolation process is predicted to remove contaminant miRNAs we anticipate higher proportions of cellular RNAs in the CS fraction compare to the S1 class. To directly test this we similarly compared expression levels of cellular RNAs stratified into different intensity tiers between the two plasma derived fractions. For all miRNA strata compared, we observe a statistically significant down regulation in abundances for cellular miRNAs in the S1 class with a p-value of <2.3e−12 (Fig. 4B). This indicates a clarification of these RNAs from the CS to the subsequently derived S1 class as a consequence of the fractionation process. Taken together these result suggests that isolation of the S1 class from the CS minimizes the levels of contaminating cellular RNA, while preserving the expression of circulating miRNA species.

### 5. Stability of expression of circulating miRNAs across biological replicates

**Figure 5:**
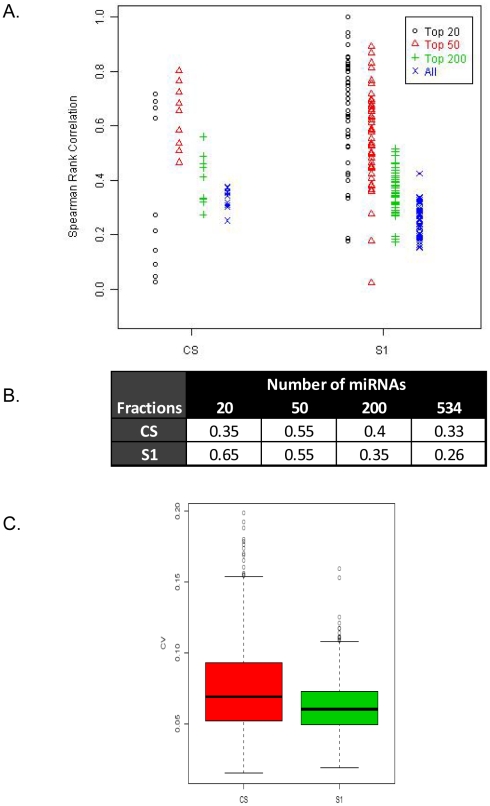
Correlation of expression levels of circulating miRNAs across different biological replicates in the CS and S1 fractions. (A) Spearman's Rank Correlation coefficients for CS and S1 fractions, across all replicates restricting to the highest expressing 20, 50, 200 or 534 (all) human miRNAs that are common to the 2 fractions. Each point on the graph represents the rank correlation values across all pair-wise combination of replicates for the category under study. (B) Mean correlation values for the CS and S1 fraction in each intensity strata. (C) Analysis of Coefficient of Variance of the CS and S1 fractions for the 534 miRNAs under study.

In principal the process of sub-fractionation, while removing contaminants may potentially impact the stability of expression of the truly circulating miRNA species. In order to assess if the population of miRNAs detected in the CS and S1 fractions display consistent patterns of expression, we analyzed both the rank order concordance of miRNAs in the CS and S1 fractions across all biological replicates and the distribution of the Coefficient of Variance (CV) between these two fractions for all miRNAs within the replicates studied. We hypothesized that conserved and stably expressed miRNAs would have non-stochastic expression levels and hence demonstrate not only high rank order correlations but also low CV estimates. In order to test this, miRNAs were selected after filtering out contaminant features and a common set of targets isolated based on average intensities from the CS and S1 fractions across multiple biological replicates. Categories of miRNAs were stratified into intensity bins containing the highest expressing 20, 50, 200 or 534 miRNAs and Spearman's Rank Correlation values computed for each category per individual fraction across all samples (Fig. 5A). Our results show modest improvement in the mean correlation values for the CS fractions across the different intensity strata (Fig. 5B). In contrast we observe a statistically significant 2.5 fold increase in correlation in the S1 fraction as the analysis is restricted from the entire 534 features to the highest expressing top 20 miRNA class (P-value of 9.624552e-18 from two-sided Student's t-test). Additionally the comparison of variance between the different fractions across all replicates also reveal a statistically significant higher variance for the CS fraction compared to the S1 isolate for the entire spectrum of 534 miRNAs (p-values of 6.138508e-13 from Student's t-test) (Fig. 5C). Taken together these results indicate that the population of miRNAs in the S1 fraction is homogenously expressed across replicates with significantly lower variability than species measured in the CS fraction. Additionally the process of fractionation of CS into S1 tends to preserve the integrity of the higher expressed miRNAs with no additional negative impact on the lower abundant species. The S1 fraction hence represents a comprehensive plasma category enriched in circulating markers that can be reproducibly detected in replicate samples for biological analysis.

### 6. Variability in expression of circulating miRNA species in cohorts of normal males and females

**Figure 6:**
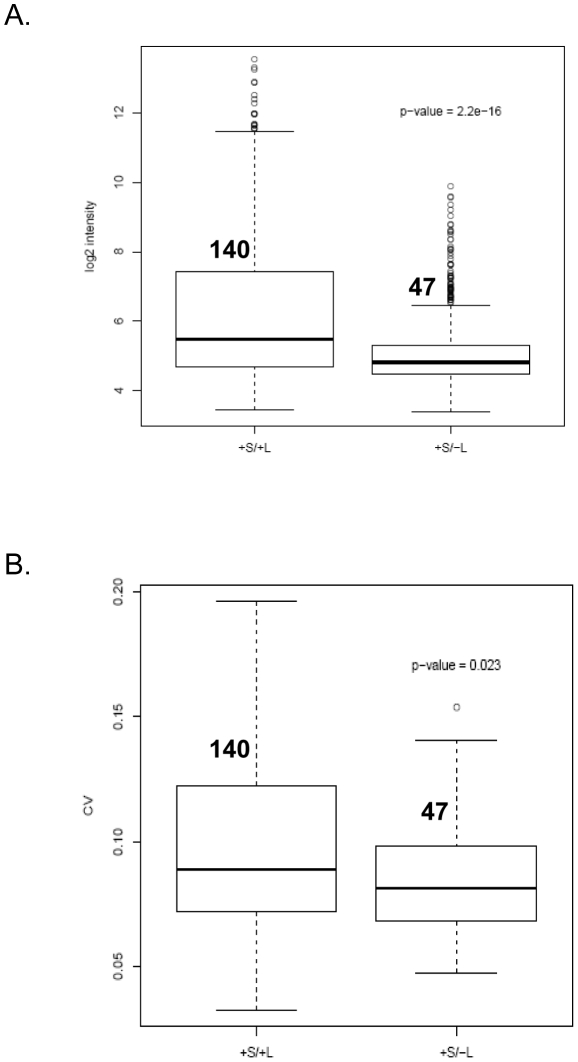
Variability of circulating miRNA expression levels in normal cohorts of male and female individuals. (A) Box plot of intensity distributions of 140 features common to both circulation and in contaminants (+S/+L) or 47 features specific only to circulation (+S/−L). The black bar represents the median of each distribution. The open circles represent the outliers. (B) Analysis of Coefficient of Variance of these two categories. P-value from two-sided Student's t-test measuring tests of significance is reported.

The intrinsic variability in expression of a biomarker is a critical determinant for understanding both its normal behavior as well as assessing diseased induced changes. To explore the expression characteristics of circulating miRNAs, we investigated both the intensity and variability of expression of markers isolated from the S1 fraction in cohorts of normal individuals. Samples extracted from 8 male and 10 female Caucasian donors were background adjusted, quantile normalized and the summarized intensities for all microRNA and background probes analyzed (Table S1 and Fig. S2). In order to ensure that markers displaying a wide range of expression values were included in this analysis, individual miRNAs were selected upon presence/absence calls and categorized based on a 50% detection threshold amongst the 18 individuals. Two categories of miRNAs were defined in the S1 fraction: (1) 140 miRNAs present in both circulation (designated as S) and those that map cumulatively to the contaminant profile derived from WBC, RBC and Leukocytes (designated as L): (+S/+L) and (2) 47 miRNAs present in circulation only: (+S/−L) (Table S3, Fig. S3). The variability in each class was assessed through Coefficient of Variance (CV) estimates. We observe a statistically significant reduction in both intensities (p-value of 2.2e-16 from two-sided Student's t-test) (Fig. 6A) and CV measurements (p-value of 0.023) (Fig. 6B) in the circulating class (+S/−L) compared to miRNAs that are co-detected both in circulation and contaminants (+S/+L). This result demonstrates that the variability in expression of circulating miRNA species in a population is reduced after removal of signatures originating from contaminating cellular miRNA profiles. Furthermore, examination of the 47 circulating miRNAs reveals greater than two-fold dynamic range of intensity for this class (Fig. S3) and harbors candidates that bear evidence of expression derived from tissues of non-hematopoietic origin. Specifically we find hsa-miR-122 to be the highest expressing circulating miRNA in our current dataset with tissue specific expression derived from the liver (Fig. S3) [21], [22]. Our analysis therefore clearly distinguishes the effects on classification and variability that arise due to varying levels of cell-derived miRNAs in samples and underscores the importance of isolation practices for the study of circulating species.

### 7. Map of differentially expressed gender specific circulating miRNAs

**Figure 7:**
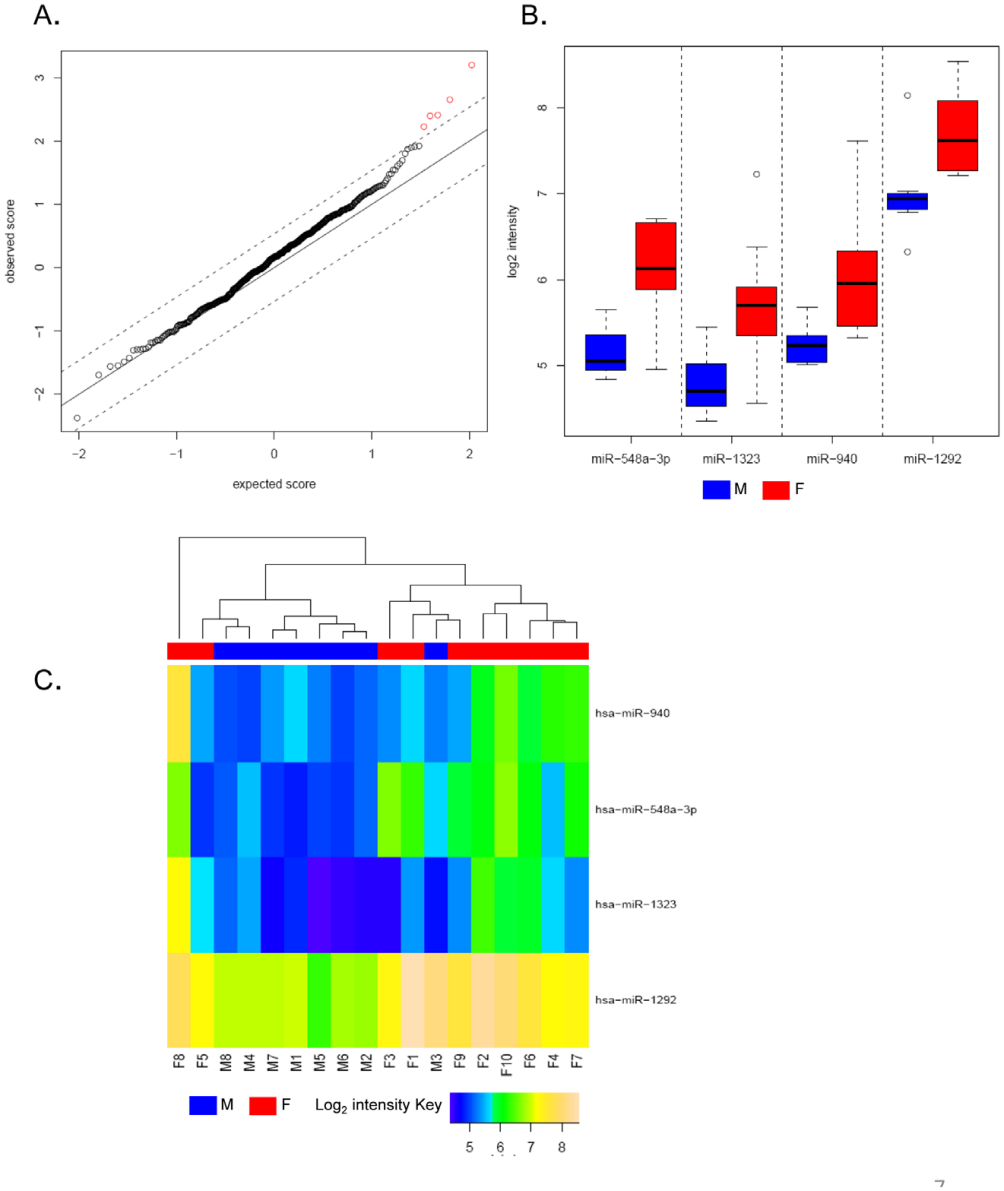
Analysis of differentially expressed miRNA species present in gender specific categories. (A) Comparison of observed versus the expected scores obtained by SAM analysis of all 534 features from 8 males and 10 Caucasian females. Each feature is represented by an open circle, and the differentially expressed features represented as red points in the graph. The dashed lines represent a FDR threshold of 5%. (B) Distributions of normalized log_2_ signal intensities of 4 differentially expressed features in males (M1–M8; blue) and females (F1–F10; red). (C) Hierarchical clustering of samples (males in blue: M1–M8 and females in red: F1–F10) based on summarized intensity values from the 4 differentially expressed circulating miRNAs. The log_2_ intensity values are shown in the bar scale.

We next wanted to determine if there were subsets of miRNAs in our dataset that demonstrated gender specific expression in populations of normal male and female individuals. To distinguish this we performed Significance Analysis of Microarrays (SAM) [23] on the entire collection of 534 miRNAs derived from 8 males and 10 female individuals after removal of contaminant features. We find a total of 5 features to be significantly differentially expressed between males and females at a False Discovery Rate of 5% (Fig. 7A). Removal of one undetected feature based on Wilcoxon - Rank Sum Test yield a subset of 4 differentially expressed circulating miRNAs present as significant gender-specific discriminators in the individuals studied (Fig. 7B and C and Fig. S4). Through an analysis of intensity distributions and q-value estimates [24], we find all of the 4 miRNAs (hsa-miR-548-3p, hsa-miR-1323, hsa-miR-940 and hsa-miR-1292) to be significantly up regulated in females with a 1.63 to 1.94 fold-change in intensity levels (Fig. S4). This fold increase has also been independently verified through quantitative PCR for two representative miRNAs (the high abundant hsa-miR-1292 and low abundant hsa-miR-1323) (Fig. S5). Interestingly, we did not detect any significantly down regulated miRNAs in this dataset. For all differential expression analyses the q-value estimates were found to be significant (Fig. 7B and Fig. S4) [24]. Expression map of these 4 miRNAs through unsupervised hierarchical clustering (Fig. 7C) show a clear separation of the two groups with the exception of 4 female individuals (F1, F3, F5 and F8) indicating that the miRNA expression profiles between the two groups were significantly different. We observe a strong and uniform expression of all up regulated miRNAs (∼log2 intensity of 6) across most individuals with particularly robust expression observed for hsa-miR-1292 (∼log2 intensity of 7.5) (Fig. 7B and C). Taken together this data characterizes profiles of differentially regulated circulating miRNAs and reveals distinct gender specific expression patterns for subsets of miRNAs in cohorts of normal male and female individuals.

## Discussion

In this study we have systematically evaluated the role of heterogeneous cellular miRNAs derived from disparate hematopoietic cells as a pre-analytic variable influencing the isolation and analysis of cell-free circulating miRNAs. Our results show that different fractionation procedures for plasma have varying degrees of efficacy in the removal of Red and White Blood Cells and as a consequence can play a major role in impairing circulating miRNA signatures. Specifically through differential centrifugation of whole blood into distinct classes of supernatants and pellet fractions we show disparate distributions of cellular miRNAs and furthermore demonstrate that the Cloudy Supernatant derived from the first spin is enriched in contaminant miRNAs compared to isolates of subsequent spins (S1 and S2) (Fig. 1). Two lines of evidence support this observation. Firstly, fractionation of the CS isolate leads to a clear separation of signal intensities between the CS derived, S1 and P1 sub-fractions (Fig. 2A). We observe a greater magnitude of signal intensity in the CS and P1 isolates compared to the S1 class, indicative of the presence of higher abundant miRNAs in these fractions. Given the cellular nature of the derivative P1 isolate, we conclude that a proportion of high abundant cellular miRNAs persist in the CS fraction that is sequestered in a pellet only as a consequence of additional centrifugation step (Fig. 2A). Secondly, inter-fraction Pearson's correlations analysis conclusively establishes a strong correspondence between the contaminants and the contaminant-rich fractions of CS and P1. This indicates an enrichment of cellular miRNAs in these classes. In contrast a relatively weaker correlation is seen for the S1 and the S1 derived, S2 and P2 fractions, suggesting that the miRNA signature of these fractions are distinct from the cellular class (Fig. 2C). We also observe that, the P1 and P2 pellet fractions are distinctive, with minimal precipitate observed in the P2 isolate. This indicates that the majority of cellular miRNAs are primarily segregated in the P1 fraction with inconsequential carryover into the P2 isolate. The residual contaminant species detected in the S1 or S2 fraction is therefore likely to be representative of cellular miRNAs that are present either due to active release from cells of hematopoietic lineage or are derived from haemolyis of cells during the isolation process.

Since we observe a high degree of similarity between the S1 and S2 isolates (Fig. 2C), we chose to focus on the S1 fraction and assess the advantages of using this fraction over the Cloudy Supernatant class. We wished to evaluate that the process of removal of contaminating miRNAs from the CS fraction did not lead to the loss of abundance or integrity of the circulating species. We examined the inter-fraction correlations of detected miRNA intensities stratified into different intensity bins and observe a strong correlation between the CS and S1 fraction indicating preservation of circulating species (Fig. 3). We also observe no significant difference in intensity distributions between stratified miRNA classes between the CS and S1 fractions supporting the homogeneity of expression of common representative miRNAs between these classes (Fig. 4A). The slightly lower intensities in the S1 fraction are likely due to loss of RNA due to additional extraction steps. Additionally our analyses also distinguish a clear reduction in expression of contaminant miRNA signatures in the S1 fraction compared to the Cloudy Supernatant class (Fig. 4B). This result conclusively demonstrates that the process of sub-fractionation enhances the specificity of circulating markers by removal of cellular miRNA species. Since both the structural and functional integrity of miRNA populations can be influenced by procedural effects, we hypothesize that one of the characteristics of specific circulating miRNA markers should be defined by the stability of expression across multiple biological samples. In order to measure reproducibility of expression we examined intensity – stratified bins of miRNAs in the CS, and S1 fractions across multiple replicates through Spearman's Rank Correlation Analysis. Additionally distribution of CV estimates between the two fractions across the profiled miRNA dynamic range was also measured. From both these analyses, we observe (a) statistically significant increase in expression level correlations across different miRNA strata in the S1 compared to the CS fraction, indicating an improved measurement of secretory miRNA population especially those of higher intensities in the S1 supernatant (Fig. 5A and B) and (b) a reduction in CV estimates for the S1 fraction compared to the CS isolate demonstrating that the variability in quantitation of circulating species is reduced as a result of sub-fractionation of CS into S1. Taken together both these results strongly affirm the consistency in miRNA signatures in the S1 supernatant fraction.

It is known that the signatures of plasma/serum miRNAs can reflect correlations to physiological or disease conditions [10]. To date 13 types of cancer have been investigated in which expression profiling of circulating miRNAs have revealed both diagnostic and prognostic utility for this class of biomarkers [11]. Since gene expression as a quantitative phenotype is known to vary within a population, in order to obtain miRNA signatures related to disease classification, it is important to evaluate the range of inter-individual variability across demographic populations. To our knowledge no studies have directly explored this variation in the context of calibrating for cellular miRNA signals. We sought to assess this variability by mapping the circulating miRNA expression profiles from the S1 fraction in healthy population of Caucasian male and female individuals both through Coefficient of Variation (CV) Analysis and Standard Deviation (SD) estimates (data not shown) (Fig. 6). Stratification of miRNAs based on detection in circulation (S) and/or contaminant (L) classes reveal an approximate loss of 66% of all detected miRNAs (93 out of 140 miRNAs that are lost between the +S/+L and the +S/−L classes) through the removal of cellular miRNA derived signatures. The proportion of this reduction is comparable to similar extensive overlaps seen between miRNA profiles derived from plasma micro vesicles and peripheral blood mononuclear cells [25]. A direct comparison of the 20 most common circulating miRNAs from healthy individuals over 5 different datasets (reviewed in [12]) reveal, that in agreement to our study at least 75% (15/20) of the reported circulating miRNA species can be mapped to our cellular miRNA signatures (Table S4). Markers that are common to both the cellular and circulating categories (+S/+L) display a significantly higher variability in both intensity and variance estimates in contrast to miRNAs that are specific only to circulation (+S/−L) (Fig. 6A and B). This analysis clearly indicates that a considerable proportion of cellular miRNAs persevere in clarified plasma preparation and moreover exhibit significant variability in expression across different individuals in a population. Cumulatively this could be reflective of either varying levels of hematopoietic cell lysis or differences in the proportion of RNA actively released into circulation from hematopoietic cells. Calibration of circulating miRNA signals for specificity though subtraction of these features allows for an improved estimate of intrinsic variability within individuals. Additionally, examination of the relationship of 47 miRNAs (Fig. 6) in the circulatory class to published lists of circulating miRNAs [26] reveal overlaps between the datasets indicating detectibility of subsets of these biomarkers across different experimental platforms (Table S3). Through literature query and search of two functional databases (miRNAorg:http://www.microrna.org and miRNAMap: http://mirnamap.mbc.nctu.edu.tw/) [27] we can find additional correspondence to tissue derived expression specificity for a subclass of these RNAs [21], [22]. Specifically we can map hsa-miR-122, hsa-miR-495, hsa-miR-34b, hsa-miR-198, hsa-miR -202, hsa-miR-510 and hsa-miR-658 to expression derived from diverse tissues of origin (Table S3), supporting the hypothesis that non-hematopoietically derived miRNAs can enter and persist in circulation. The preservation of these species in a cell-free environment is intriguing. Emerging evidence point towards sequestration of secretory miRNAs in sub-cellular particles such as exosomes [19] and microvesicles [25]. This association provides a model where packaging of miRNAs allow both for survival in a catalytic extracellular environment and in addition provide a route for trafficking of these species through circulation [19] to satisfy a range of regulatory requirements. Given that the fractionation protocol utilized in this study preserves the integrity of these sub-cellular particles in the S1 fraction, it is interesting to speculate on the involvement of these particles in the potential localization of the circulatory miRNAs identified in this study.

Gender specific differences are known to play a role not only in the type and susceptibility of diseases but also affect the response to therapeutic treatments [28]. In order to assess if the expression of circulating miRNAs demonstrated any gender specificity we sought to explore miRNAs signatures in random cohorts of normal male and female individuals. Through Significance Analysis of Microarrays (SAM) we distinguished 4 circulating miRNAs that are differentially expressed in a gender specific manner. We find all of these miRNAs to be significantly up regulated by 63–95% in females and detect no miRNAs which display a reduction in expression (Fig. 7B and C and Fig. S4). We can verify the fold differences for a subset of these candidates through qPCR analyses (Fig. S5) and additionally find correlation of expression of one out of these four miRNAs to sex-specific tissue. Specifically we show that the strong female specific expresser: hsa-miR-940 to be detected in the cervix [29] (Fig. S4). In addition through a search of the Genes-to-Systems Breast Cancer Database (http://www.itb.cnr.it/breastcancer/) we find evidence of interaction of all the 4 miRNAs with gene targets such as E2F1, E2F3, DAPK1 and others implicated to be altered in breast cancer cells (Fig. S4). Taken together these results provide functional indications for gender and disease associated role of these miRNAs. Structurally, we find majority of the differentially expressed circulating miRNA species (75% or 3/4) originate from unannotated intergenic regions with only hsa-miR-1292 mapping to the intron of a gene. Although the biological roles of these specific miRNAs are obscure, these results thus provide clear evidence for differential expression of circulating miRNAs in a gender specific manner. Additionally, correlation of expression to sex-specific tissues for a subset of these transcripts, provide a compelling rationale for a physiological role of these miRNAs in delineating biological differences between the genders.

Several studies have explored the utility and analytical procedures for the study of circulating miRNAs. Although the precise functions and mechanism of action of these RNAs remain undetermined [11], [14] accumulating evidence in the form of qPCR and sequencing data [10], [30] from a variety of conditions ranging from malignancies to pregnancy (reviewed in [12] and [17]) has begun to delineate the diversity of this class of miRNAs as molecular biomarkers correlating expression with physiological states. To our knowledge our results address for the first time comprehensive global signatures of bona fide circulating miRNA species in context of cellular miRNA expression in cohorts of normal individuals. By segregating the specificity of measured signals into cell-free and cellular miRNAs we demonstrate the heterogeneity in expression of fluid-derived biomarker signatures. Additionally, our data provides categories of miRNAs that can help distinguish circulating miRNAs from cellular counterparts. The resultant classifications of these biomarkers into cell-associated and extracellular circulating species in part help mitigate the inherent ambiguity in the interpretation of these profiles. Taken together these results provide a comprehensive map of the circulatory miRNA environment in normal individuals and provide compelling motivation to explore such landscapes in detail in pathological conditions, where circulating miRNA signatures may be further confounded by disease specific over-expression of hematopoietically derived miRNAs. As formal recommendations for the study of secretory biomarkers evolve, we anticipate that analysis of circulating miRNAs in such class-specific manner would advance both the detection and quantitation strategies utilized for global investigations of miRNA biomarker study.

## Materials and methods

### 1. Ethics statement

Anonymized human blood samples were purchased from Stanford Blood Center (http://bloodcenter.stanford.edu/). Written consent was obtained from all patients prior to obtaining samples. Stanford Blood Center is a division of the Stanford University School of Medicine and complies with all ethical guidelines of their Institutional Review Board. At Affymetrix all applicable OSHA guidelines (http://www.osha.gov/) for working with human blood samples were strictly adhered to.

### 2. Fractionation of plasma from whole blood and isolation of miRNA

Whole Blood (5–10 mL) was collected from healthy male and female Caucasian donors; age matched within 25 years in Sodium EDTA tubes (Table S1). The samples were stored on ice and processed within 4 hours of draw. All centrifugation steps were performed at 4°C. All samples were initially spun at 1700 g for 10 minutes to separate the plasma from the Buffy Coat and Red Blood Cells. Leukocytes were separately isolated from whole blood stabilized with yeast tRNA (Ambion) and fractionated through Ficoll-Hypaque gradients. The plasma (designated as the Cloudy Supernatant) was then re-centrifuged at 2000 g for 10 minutes to obtain the Supernatant 1 (S1) and Pellet 1 (P1) fractions. F or the fractionation studies the S1 fraction was additionally centrifuged at 12000 g for 10 minutes to generate the Supernatant 2 (S2) and Pellet 2 (P2) fractions. All the pellet fractions were washed once with 1xPBS and then reconstituted in 0.5 ml 1xPBS. Similarly the Red Blood Cell fraction was washed twice with 3 x volumes of 1xPBS and the pellet collected. To all fractions 3 volumes of Trizol-LS reagent (Invitrogen) and 10 ug/mL of Yeast Total Carrier RNA (Ambion) were added to stabilize the samples. Samples were isolated through multiple organic extractions using 0.3 volume of chloroform followed by phenol chloroform extractions. Total RNA including miRNA was purified from the aqueous phase using 1.25 volume of ethanol and eluted through the mirVANA columns (Ambion).

### 3. Labeling and hybridization of plasma samples to the Affymetrix miRNA Arrays

Total RNA ranging in concentration from 500–1000 ng were labeled using the Genisphere HSR labeling kit (P/N HSR30FTA) and hybridized overnight to the Affymetrix Genechip miRNA array (P/N 901326) (Table S1). The arrays were washed and stained using standard Affymetrix protocols and scanned using the Affymetrix GCS 3000 7G Scanner. Feature intensities were extracted using the miRNA_1-0_2xgain library files.

### 4. Analysis of RNA integrity

Total RNA (50-100 ng) was analyzed using the Agilent small RNA kit in the 2100 Bionalyzer using manufacturer's recommendations. Additionally for the leukocytic and RBC samples ∼300–400 ng of RNA was resolved on a 15% TBE-UREA gel to demonstrate the integrity of total RNA. This is especially relevant for samples (i.e. Leukocytes and WBC) where visualization of small RNA bands is challenging. Due to limiting material from one sample derived from the CS fraction (20100915_RD_Fractionation_A_Plasma_RNA_800ng_B1_T1.cel) a migration analysis was not possible. To estimate the integrity of this sample, Pearson's correlation of the intensity levels of all normalized human miRNAs for this specific sample versus all other CS samples in this plasma class was performed. The computed correlation value is 0.93 with an R square value of 0.86 indicating a high performance of this sample in relation to the rest (data not shown).

### 5. Quantitative RT-PCR

Assays to quantify differential expression of gender specific miRNAs were performed using the ABI 7500 real-time PCR instrument and miScript quantitative PCR System (Qiagen). Pooled Male (M1–M8) and Female (F1–F10) samples were used for reverse transcription. Approximately 4 ng and 10 ng of cDNA were used to measure hsa-miR-1292 and hsa-miR-1323 levels, respectively. The normalizer used in this data was U6. Similar results are obtained when using miRNAs that are not found to be differentially expressed by SAM analysis as normalizers (data not shown). Due to greater sensitivity of the qPCR assay the detected fold change is on an average 1.3 to 2.5 fold higher than the array data.

### 6. Datasets

All data described in this manuscript are MIAME compliant and have been deposited in NCBI's Gene Expression Omnibus Database (http://www.ncbi.nlm.nih.gov/geo/). The data series is accessible through GEO Series accession number GSE27256: http://www.ncbi.nlm.nih.gov/geo/query/acc.cgi?acc=GSE27256.

### 7. Analysis of the miRNA data

#### Data Preprocessing

The workflow for data preprocessing consisted of extraction of intensities for each individual feature followed by detection calls based on Wilcoxon-Rank Sum test, background subtraction based on GC content of anti-genomic probes, transformation of values through addition of a small constant (value 16), quantile normalization and finally median summarization of all probe sets for each feature. The detection and background adjustment were done via Affymetrix miRNA QC Tool and the rest of workflow was performed under R programming environment (www.r-project.org) [31]. All reported intensity data are log_2_ transformed. All p-values are calculated by two-sided Student's t-test.

#### Generation of contaminant features list

For determining the list of miRNAs that were present in the contamination class, we first determined the number of detected miRNAs in each of the 3 contaminant categories of Red Blood Cells (RBC), White Blood Cells (WBC) and Leukocytes (L). A 100% detection criterion (i.e. present in all of the samples in each category) was applied for selecting individual miRNAs. A union of all the miRNAs present in these three classes was then taken to obtain 313 miRNAs that are co-detected in all these 3 contaminant classes (Table S2).

#### Analysis of Fractionation Data

For comparison of signal distributions (Fig. 2A) and rank correlations (Fig. 2C, 3, 4 and 5) between different plasma (CS, S1, S2, P1 and P2) and cellular categories (L, W and R), non-normalized data from each fraction was analyzed to preserve the individual distributions. For inter- and intra-fraction correlation analyses the average intensities for all the 847 human miRNAs (Fig. 2C) or 534 human miRNAs that can be counted after removal of contaminant features (Figs. 3, 4 and 5) were first computed across all samples within an individual fraction under study. The average intensities were then sorted and miRNAs binned into different intensity strata. Spearman's rank correlation coefficients were calculated for a given set of features in each intensity bin for comparison between or within a particular set of fractions.

#### Analysis of variability Data

For exploration of inter-individual variability in healthy cohorts of 8 males and 10 females, all individual samples were quantile normalized together and the numbers of detected miRNAs selected based on the Wilcoxon-Rank Sum test. Features were then filtered for contaminant miRNAs and counted based on a 50% detection threshold for the population under study (i.e. present in at least 9 out of the 18 individuals). Subsequently, selected miRNAs were stratified into 2 classes based on presence (+) or absence (−) of detected features in contaminants (derived from WBC, RBC and Leukocytes and designated as L) or circulation (S). The number of miRNAs in the two categories selected were: (a) +S/+L: with 140 features and (b) +S/−L: with 47 features. For each of these classes, intensity and variability was computed including measurements from all individuals. Variability estimates were done through either Coefficient of Variation analysis (Fig. 6B) or Standard Deviation estimates (data not shown).

### 8. Gender specific differential expression and Hierarchical clustering of intensity data

For exploration of gender specific differential expression, normalized summarized intensities from 8 males and 10 females were analyzed using Significance Analysis of Microarray data (SAM) [32]. All 534 features were included in this analysis after removal of contaminant miRNAs and features with significant differential expression levels (DE) detected at a false discovery rate (FDR) of 5%. Expected scores were calculated though 1000 permutations in SAM. A total of 5 DE features were determined which were subsequently filtered for detection calls and miRNAs not detected in any samples were removed (Fig. 7A). This process resulted in the elimination of hsa-miR-1181. From this analysis a total of 4 up-regulated features were distinguished in females (Fig.7B, C and Fig. S4). Statistical significance of differential expression was measured by the q-value which is an *estimate* of FDR and is usually a number greater than 0. In our dataset due to the limiting sample size (8 males and 10 females) we observe that the test statistic of a feature is more extreme than all observed permutations. This indicates that the *estimated* FDR, by declaring this feature as significant, is equivalent to 0. All differentially expressed features were further selected for clustering to delineate relationships between the two sexes. The unsupervised Hierarchical clustering algorithm in the R “hclust” function was used with Euclidean distance matrix and complete-linkage agglomeration. The heat map was generated by the R “gplots” package (Fig. 7C). All gender specific features were mapped to the April 2010 mirBASE release 16 to get chromosomal and genomic locations.

## Supporting Information

Figure S1 (**A–I**)**.** Agilent 2100 Bioanalyzer and PAGE analysis of RNA integrity for samples used in this study.
(TIF)

Figure S2 Box plot of signal intensity distribution of human miRNAs (white) and background probes (red) for 8 males and 10 females after background subtraction, quantile normalization and median summarization.
(TIF)

Figure S3 Box plot of signal intensity distribution of 47 human miRNAs specific only to circulation (+S/−L) in healthy cohorts of 8 male and 10 female individuals.
(TIF)

Figure S4 Table of statistically significantly differentially expressed miRNA features in females compared to males based on SAM analysis. The “Score” represents the modified t-test statistics calculated by SAM. The “Fold Change” denotes the ratios of the mean intensity in female samples over male samples. Tissue specific expression is derived from mirBASE, miRNAorg or miRNAmap databases.
(TIF)

Figure S5 (**A–B**)**.** Expression levels of hsa-miR-1292 and hsa-miR-1323 (n = 4, *P values <0.01) measured by qPCR. The p values are calculated based on a Student's t-test of the replicate 2∧(−ΔCt) values for each miRNA in the control group (males) and test groups (females).
(TIF)

Table S1 List of experimental datasets.
(XLSX)

Table S2 List of 313 miRNAs in the contamination list. Un-normalized intensity values and detection calls from each sample are represented.
(XLSX)

Table S3 List of 140 miRNAs common to both circulation and in contaminants (+S/+L) or 47 miRNAs specific only to circulation (+S/−L). Normalized intensity values and detection calls from each sample are represented.
(XLSX)

Table S4 Overlap of 20 most common circulating miRNAs from healthy individuals and 140 miRNAs present in the +S/+L category.
(XLS)
