# Supplementary figures and images for: Impact of Cellular miRNAs on Circulating miRNA Biomarker Signatures
Source: PLoS One. 2011 Jun 17;6(6):e20769. doi: 10.1371/journal.pone.0020769 (PMC3117799; doi:10.1371/journal.pone.0020769)

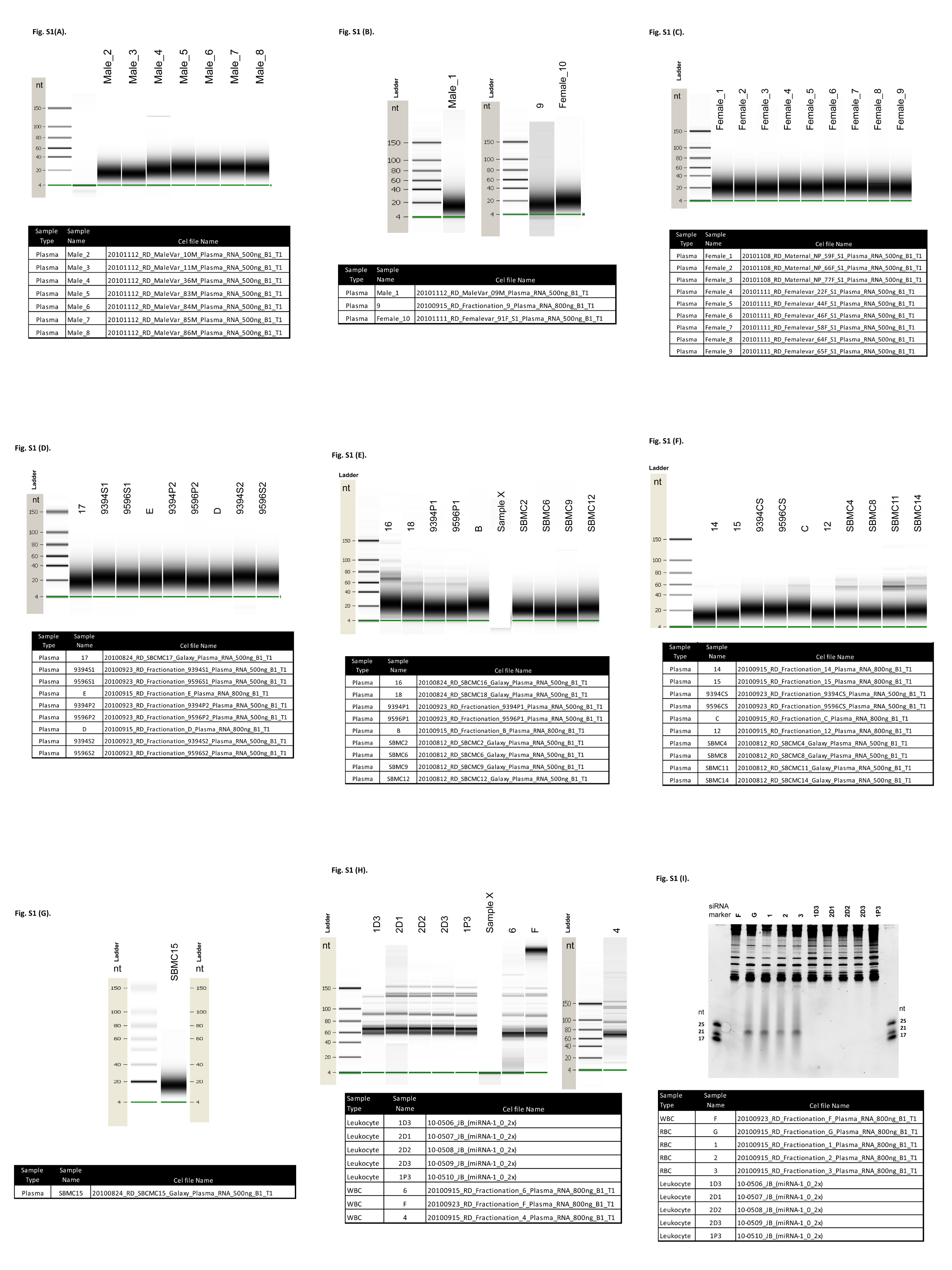

Supplement: Figure S1 — (A–I). Agilent 2100 Bioanalyzer and PAGE analysis of RNA integrity for samples used in this study. (TIF) [file pone.0020769.s001.tif]

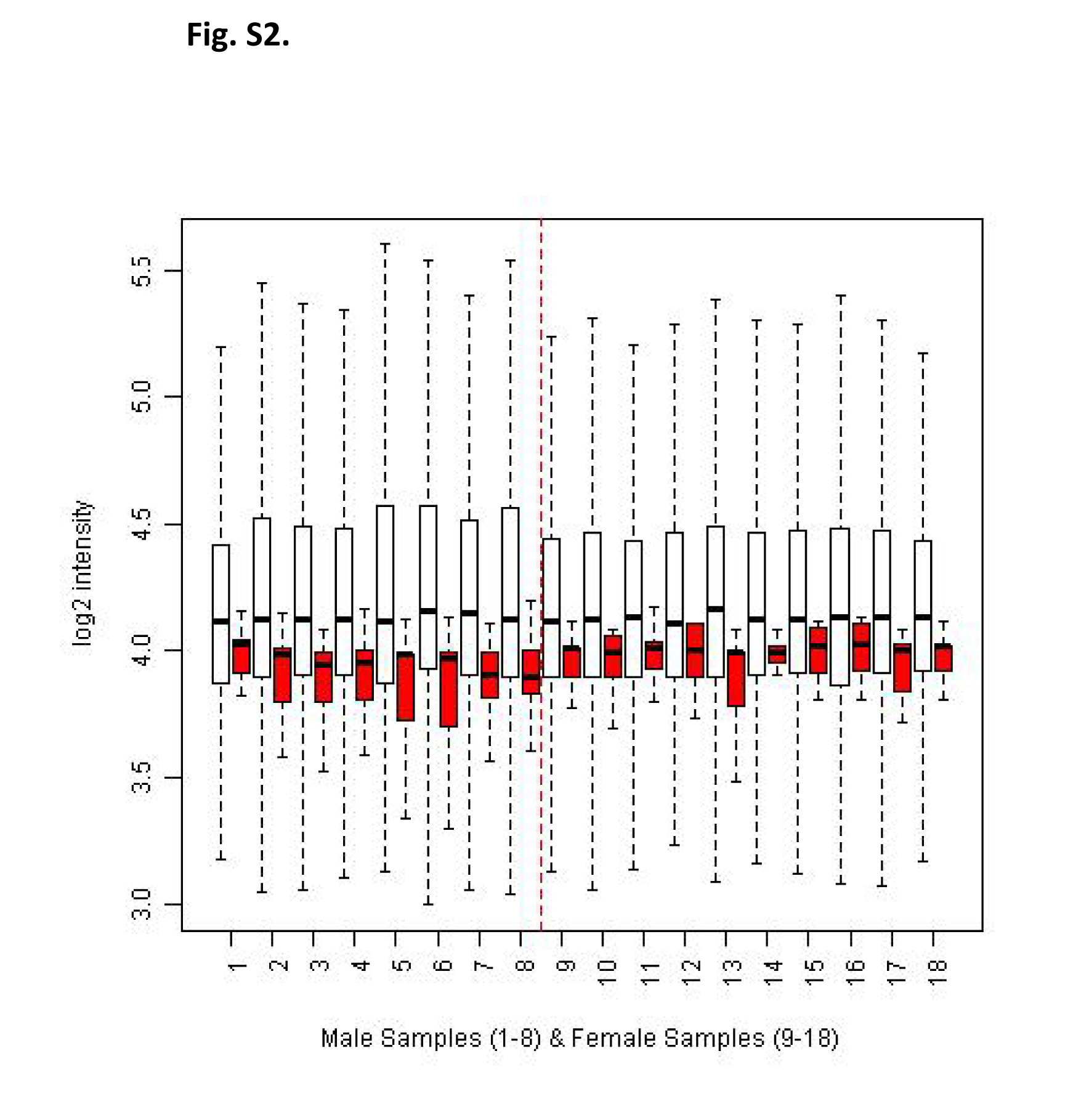

Supplement: Figure S2 — Box plot of signal intensity distribution of human miRNAs (white) and background probes (red) for 8 males and 10 females after background subtraction, quantile normalization and median summarization. (TIF) [file pone.0020769.s002.tif]

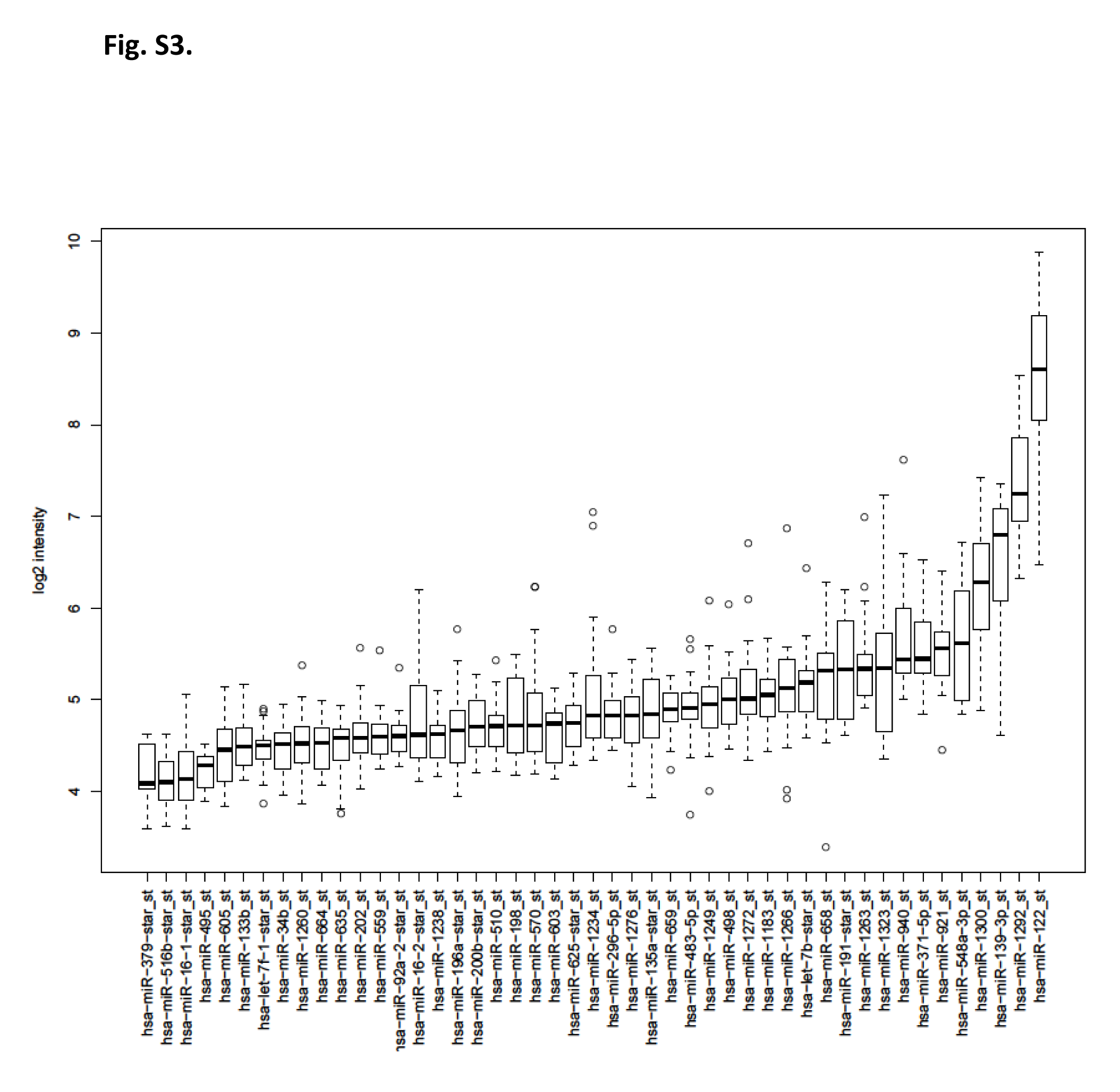

Supplement: Figure S3 — Box plot of signal intensity distribution of 47 human miRNAs specific only to circulation (+S/−L) in healthy cohorts of 8 male and 10 female individuals. (TIF) [file pone.0020769.s003.tif]

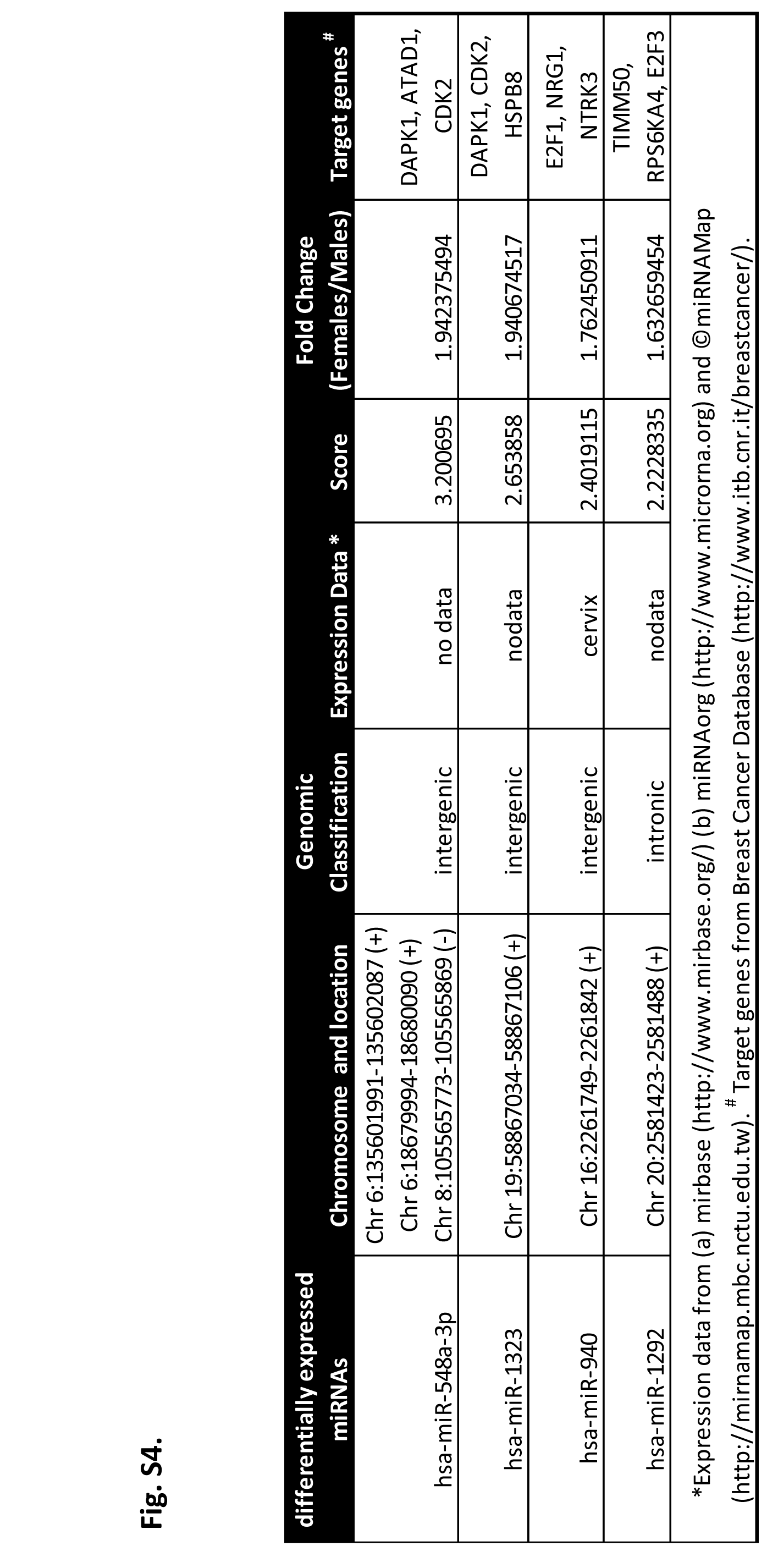

Supplement: Figure S4 — Table of statistically significantly differentially expressed miRNA features in females compared to males based on SAM analysis. The “Score” represents the modified t-test statistics calculated by SAM. The “Fold Change” denotes the ratios of the mean intensity in female samples over male samples. Tissue specific expression is derived from mirBASE, miRNAorg or miRNAmap databases. (TIF) [file pone.0020769.s004.tif]

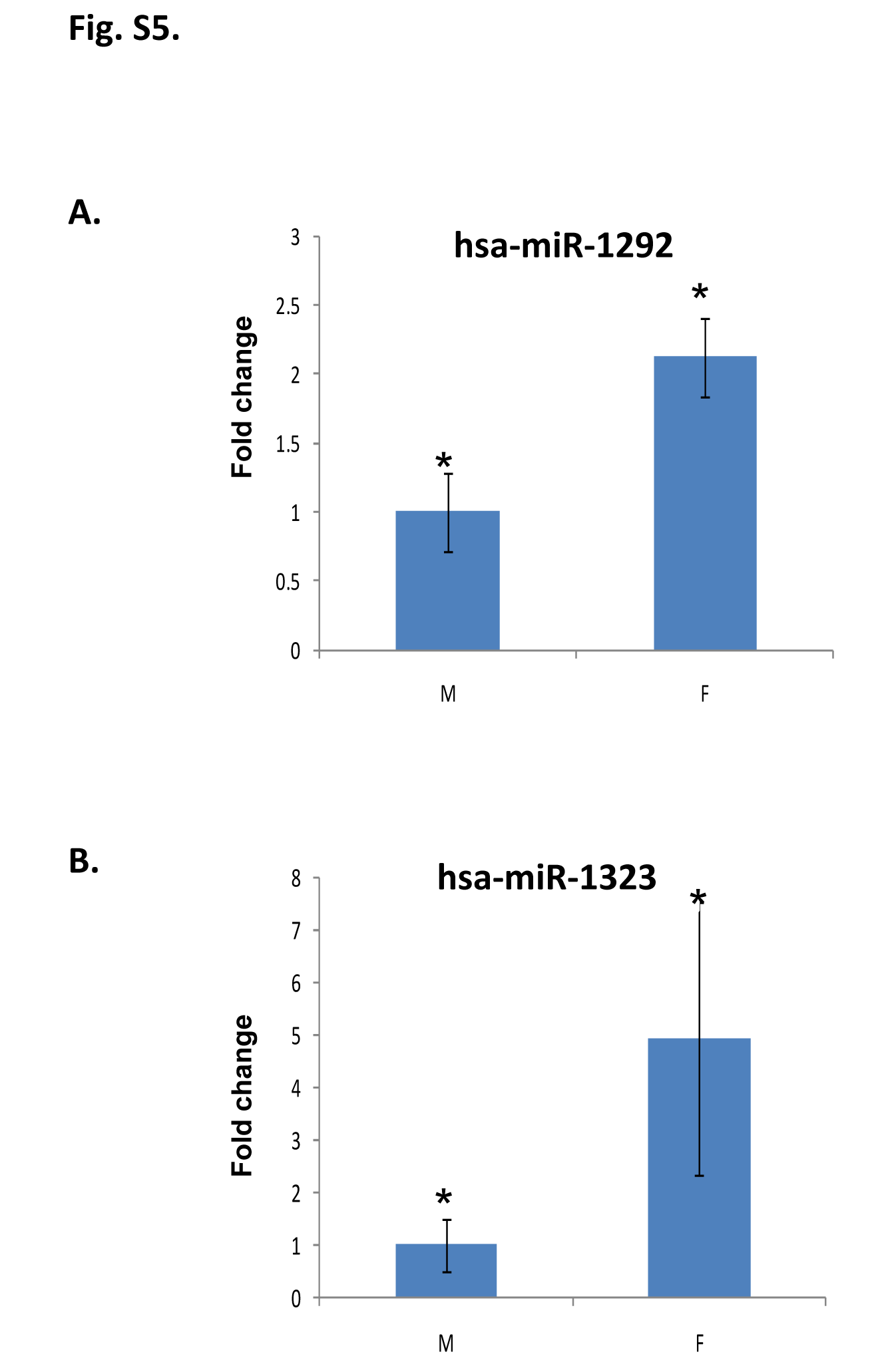

Supplement: Figure S5 — (A–B). Expression levels of hsa-miR-1292 and hsa-miR-1323 (n = 4, *P values <0.01) measured by qPCR. The p values are calculated based on a Student's t-test of the replicate 2∧(−ΔCt) values for each miRNA in the control group (males) and test groups (females). (TIF) [file pone.0020769.s005.tif]
